# A new proof of the generalized Hamiltonian–Real calculus

**DOI:** 10.1098/rsos.160211

**Published:** 2016-09-14

**Authors:** Dongpo Xu, Hua Gao, Danilo P. Mandic

**Affiliations:** 1 School of Mathematics and Statistics, Northeast Normal University, Changchun 130024, People’s Republic of China; 2 College of Science, Harbin Engineering University, Harbin 150001, People’s Republic of China; 3 Department of Electrical and Electronic Engineering, Imperial College London, SW7 2AZ London, UK

**Keywords:** GHR calculus, product and chain rules, duality relationship, widely linear quaternion least mean square

## Abstract

The recently introduced generalized Hamiltonian–Real (GHR) calculus comprises, for the first time, the product and chain rules that makes it a powerful tool for quaternion-based optimization and adaptive signal processing. In this paper, we introduce novel dual relationships between the GHR calculus and multivariate real calculus, in order to provide a new, simpler proof of the GHR derivative rules. This further reinforces the theoretical foundation of the GHR calculus and provides a convenient methodology for generic extensions of real- and complex-valued learning algorithms to the quaternion domain.

## 1. Introduction

Quaternions were introduced by William Hamilton in 1843, as an associative but not commutative algebra over reals, and have been used in many fields, such as physics, computer graphics and signal processing.

Because of the non-commutativity of quaternion product, definitions of quaternion derivative are very different from those for real and complex derivatives. For example, Sudbery [1] establishes that only linear quaternion functions fulfil the requirements of traditional derivative definition. However, such a derivative is too stringent for practical optimization problems, whereby the cost functions are often real-valued and therefore non-analytic. In order to relax the derivative condition, the recently introduced Hamiltonian–Real (HR) calculus [2] deals with both analytic and non-analytic quaternion functions within a unified framework. However, the HR calculus does not comprise effective derivative rules (product, chain) for dealing with complicated derivation operations in practical problems. By exploiting quaternion rotation, the recently introduced generalized HR (GHR) calculus embarks upon the HR calculus to provide the powerful product and chain derivative rules that facilitate the calculation of quaternion gradient and Hessian in quaternion optimization problems [3]. In particular, we note that the product rule is a distinctive feature of the functional calculi. However, the original proof of the product and chain rules for the GHR calculus is quite involved and difficult to understand for non-experts.

The aim of this paper is to further elucidate the main ideas of the GHR calculus, and to give simpler proofs of the main results in [3]. The new proof is based on the duality between the GHR calculus and multivariate real calculus. One of the advantages of such an approach is that it allows for a direct treatment of quaternion-valued functions, without an intermediate transition to real functions. Based on the so-introduced relationships, we illustrate the effectiveness of such an approach by providing a dual version of the widely linear quaternion least mean square (WL-QLMS) adaptive learning algorithm [4,5], and show that it produces the same output as the primal WL-QLMS, but with a reduced computational complexity.

## 2. Preliminaries

In the following, we state some basic definitions of the GHR calculus [3,6,7].

**Definition 2.1 (Quaternion rotation [8])**

For any quaternion *q*, the quaternion rotation is defined as

2.1 $$q^{\mu }≜\mu q\mu^{-1},$$

where *μ* is any non-zero quaternion.

**Definition 2.2 (Real-differentiability [1])**

A function *f*(*q*)=*f*_*a*_(*q*)+*if*_*b*_(*q*)+*jf*_*c*_(*q*)+ *kf*_*d*_(*q*) is called real differentiable if its real-valued components *f*_*a*_(*q*), *f*_*b*_(*q*), *f*_*c*_(*q*) and *f*_*d*_(*q*) are differentiable functions with respect to four real components *q*_*a*_,*q*_*b*_,*q*_*c*_ and *q*_*d*_ of a quaternion variable *q*=*q*_*a*_+*iq*_*b*_+*jq*_*c*_+*kq*_*d*_.

**Definition 2.3 (The GHR derivatives [3])**

If f:H→H is real-differentiable, then, the GHR derivatives of the function *f* with respect to *q*^*μ*^ and *q*^μ*^ (μ≠0,μ∈H) are defined as

2.2 $$\begin{array}{rl} \frac{\partial f}{\partial q^{\mu }} & =\frac{1}{4}\left(\frac{\partial f}{\partial q_{a}}-\frac{\partial f}{\partial q_{b}}i^{\mu }-\frac{\partial f}{\partial q_{c}}j^{\mu }-\frac{\partial f}{\partial q_{d}}k^{\mu }\right)\\ \;\mathrm{and}\;\;\frac{\partial f}{\partial q^{\mu *}} & =\frac{1}{4}\left(\frac{\partial f}{\partial q_{a}}+\frac{\partial f}{\partial q_{b}}i^{\mu }+\frac{\partial f}{\partial q_{c}}j^{\mu }+\frac{\partial f}{\partial q_{d}}k^{\mu }\right) \end{array}\},$$

where *q*=*q*_*a*_+*iq*_*b*_+*jq*_*c*_+*kq*_*d*_, quaternion components $q_{a},q_{b},q_{c},q_{d}\in R$, and ∂*f*/∂*q*_*a*_, ∂*f*/∂*q*_*b*_, ∂*f*/∂*q*_*c*_ and ∂*f*/∂*q*_*d*_ are the partial derivatives of *f* with respect to *q*_*a*_, *q*_*b*_, *q*_*c*_ and *q*_*d*_, whereas the set {1,*i*^*μ*^,*j*^*μ*^,*k*^*μ*^} is an orthogonal basis of H.

**Lemma 2.4 (Duality of quaternions and real vectors [9,10])**

*For any quaternion* *q*=*q*_*a*_+*iq*_*b*_+*jq*_*c*_+*kq*_*d*_, *where* $q_{l}\in R$, *l*∈{*a*,*b*,*c*,*d*}, *the relationship between the augmented quaternion vector* $q^{a}={\left(q,q^{i},q^{j},q^{k}\right)}^{T}\in H^{4}$ *and its quadrivariate real vector counterpart* $q^{r}={\left(q_{a},q_{b},q_{c},q_{d}\right)}^{T}\in R^{4}$ *is given by* $q^{a}=Aq^{r}$, *where*

2.3 $$A=\left(\begin{array}{cccc} 1 & i & j & k\\ 1 & i & -j & -k\\ 1 & -i & j & -k\\ 1 & -i & -j & k \end{array}\right),\;A^{-1}=\frac{1}{4}A^{H}$$

*and* (⋅)^*H*^ *denotes the quaternion conjugate transpose*.

**Lemma 2.5 (Duality of quaternion gradients and real gradient vectors [7])**

*For a real-differentiable function* f:H→R, *the relation between the quadrivariate real gradient vector* $\nabla_{q}^{r}f={\left(\partial f/\partial q_{a},\partial f/\partial q_{b},\partial f/\partial q_{c},\partial f/\partial q_{d}\right)}^{T}$ *and the augmented quaternion gradient vector* $\nabla_{q^{*}}^{a}f=(\partial f/\partial q^{*},\partial f/\partial q^{i*},\partial f/\partial q^{j*}$, ∂*f*/∂*q*^k*^)^*T*^ *is given by* $\nabla_{q}^{r}f=A^{H}\nabla_{q^{*}}^{a}f$.

**Lemma 2.6 (Duality of quaternion and real Jacobian matrices)**

*For a real-differentiable function* f:H→H, *the relation between the quadrivariate real Jacobian matrix* $J_{q}^{r}(f)$ *and the augmented quaternion Jacobian matrix* $J_{q}^{a}(\;f)$ *is given by* $J_{q}^{r}(\;f)=A^{-1}J_{q^{\mu }}^{a}(\;f)A^{\mu }$, *where*

2.4 $$J_{q}^{r}(f)=\left(\begin{array}{cccc} \frac{\partial f_{a}}{\partial q_{a}} & \frac{\partial f_{a}}{\partial q_{b}} & \frac{\partial f_{a}}{\partial q_{c}} & \frac{\partial f_{a}}{\partial q_{d}}\\ \frac{\partial f_{b}}{\partial q_{a}} & \frac{\partial f_{b}}{\partial q_{b}} & \frac{\partial f_{b}}{\partial q_{c}} & \frac{\partial f_{b}}{\partial q_{d}}\\ \frac{\partial f_{c}}{\partial q_{a}} & \frac{\partial f_{c}}{\partial q_{b}} & \frac{\partial f_{c}}{\partial q_{c}} & \frac{\partial f_{c}}{\partial q_{d}}\\ \frac{\partial f_{d}}{\partial q_{a}} & \frac{\partial f_{d}}{\partial q_{b}} & \frac{\partial f_{d}}{\partial q_{c}} & \frac{\partial f_{d}}{\partial q_{d}} \end{array}\right)\;\mathrm{and}\;J_{q^{\mu }}^{a}(f)=\left(\begin{array}{cccc} \frac{\partial f}{\partial q^{\mu }} & \frac{\partial f}{\partial q^{\mu i}} & \frac{\partial f}{\partial q^{\mu j}} & \frac{\partial f}{\partial q^{\mu k}}\\ \frac{\partial f^{i}}{\partial q^{\mu }} & \frac{\partial f^{i}}{\partial q^{\mu i}} & \frac{\partial f^{i}}{\partial q^{\mu j}} & \frac{\partial f^{i}}{\partial q^{\mu k}}\\ \frac{\partial f^{j}}{\partial q^{\mu }} & \frac{\partial f^{j}}{\partial q^{\mu i}} & \frac{\partial f^{j}}{\partial q^{\mu j}} & \frac{\partial f^{j}}{\partial q^{\mu k}}\\ \frac{\partial f^{k}}{\partial q^{\mu }} & \frac{\partial f^{k}}{\partial q^{\mu i}} & \frac{\partial f^{k}}{\partial q^{\mu j}} & \frac{\partial f^{k}}{\partial q^{\mu k}} \end{array}\right)$$

*with* *f*(*q*)=*f*_*a*_(*q*)+*if*_*b*_(*q*)+*jf*_*c*_(*q*)+*kf*_*d*_(*q*), *q*=*q*_*a*_+*iq*_*b*_+*jq*_*c*_+*kq*_*d*_ *and* $q_{l}\in R,f_{l}(q)\in R$, *l*∈{*a*,*b*,*c*,*d*}.

**Proof.**

The proof follows directly from definition 2.3. ▪

**Lemma 2.7 (Duality of quaternion and real Hessian matrices [7])**

*For a real-differentiable function* f:H→R, *the relation between the quadrivariate real Hessian matrix* $H_{q}^{r}(f)$ *and the augmented quaternion Hessian matrix* $H_{q}^{a}(f)$ *is given by* $H_{q}^{r}(f)=A^{H}H_{q}^{a}(f)A$.

## 3. Main results

**Theorem 3.1 (Chain rule of GHR derivatives)**

*Let* S⊆H *and let* g:S→H *be real-differentiable at an interior point q of the set S. Let* T⊆H *be such that g(q)∈T for all q∈S. Assume that* f:T→H *is real-differentiable at an interior point g(q)∈T. Then, the composite function h(q)=f(g(q)) satisfies the following chain rule
*

3.1 $$\frac{\partial h(q)}{\partial q^{\mu }}=\frac{\partial f}{\partial g}\frac{\partial g}{\partial q^{\mu }}+\frac{\partial f}{\partial g^{i}}\frac{\partial g^{i}}{\partial q^{\mu }}+\frac{\partial f}{\partial g^{j}}\frac{\partial g^{j}}{\partial q^{\mu }}+\frac{\partial f}{\partial g^{k}}\frac{\partial g^{k}}{\partial q^{\mu }},$$

*where* μ∈H,μ≠0*, ∂f/∂g*^*ν*^*=∂f(g)/∂g*^*ν*^ *(ν=1,i,j,k) are the GHR derivatives.*

**Proof.**

Using the chain rule of multivariate real calculus, we have

3.2 $${\text{J}}_{q}^{r}(h)={\text{J}}_{g}^{r}(h){\text{J}}_{q}^{r}(g).$$

Multiplying both sides of (3.2) by **A** and (**A**^*μ*^)^−1^, we have

3.3 $$\text{A}{\text{J}}_{q}^{r}(h){\left({\text{A}}^{\mu }\right)}^{-1}=\text{A}{\text{J}}_{g}^{r}(h){\text{ A}}^{-1}\text{A}{\text{J}}_{q}^{r}(g){\left({\text{A}}^{\mu }\right)}^{-1}.$$

Using lemma 2.6 further yields

3.4 $${\text{J}}_{q^{\mu }}^{a}(h)={\text{J}}_{g}^{a}(h){\text{J}}_{q^{\mu }}^{a}(g).$$

Upon extracting the top left entry (i.e. (1,1)-entry) of the matrix equation (3.4), we arrive at

3.5 $$\frac{\partial f(g(q))}{\partial q^{\mu }}=\frac{\partial f(g)}{\partial g}\frac{\partial g(q)}{\partial q^{\mu }}+\frac{\partial f(g)}{\partial g^{i}}\frac{\partial g^{i}(q)}{\partial q^{\mu }}+\frac{\partial f(g)}{\partial g^{j}}\frac{\partial g^{j}(q)}{\partial q^{\mu }}+\frac{\partial f(g)}{\partial g^{k}}\frac{\partial g^{k}(q)}{\partial q^{\mu }}.$$

This completes the proof of theorem 3.1. ▪

**Theorem 3.2 (Product rule of GHR derivatives)**

*If the functions* f,g:H→H *are real-differentiable, then the derivative of their product fg satisfies the following product rule
*

3.6 $$\frac{\partial (fg)}{\partial q^{\mu }}=f\frac{\partial g}{\partial q^{\mu }}+\frac{\partial f}{\partial q^{g\mu }}g,$$

*where* μ∈H,μ≠0, *∂f/∂q*^*gμ*^ *can be obtained by replacing μ with gμ in definition *2.3.

**Proof.**

Denote *h*(*q*)=*f*(*q*)*g*(*q*), *f*=*f*_*a*_+*if*_*b*_+*jf*_*c*_+*kf*_*d*_ and *g*=*g*_*a*_+*ig*_*b*_+*jg*_*c*_+*kg*_*d*_. Then,

3.7 $${\text{h}}^{r}=\left(\begin{array}{c} h_{a}\\ h_{b}\\ h_{c}\\ h_{d} \end{array}\right)=\left(\begin{array}{c} f_{a}g_{a}-f_{b}g_{b}-f_{c}g_{c}-f_{d}g_{d}\\ f_{a}g_{b}+f_{b}g_{a}+f_{c}g_{d}-f_{d}g_{c}\\ f_{a}g_{c}-f_{b}g_{d}+f_{c}g_{a}+f_{d}g_{b}\\ f_{a}g_{d}+f_{b}g_{c}-f_{c}g_{b}+f_{d}g_{a} \end{array}\right).$$

Using the product rule of real calculus yields

3.8 $${\text{J}}_{q}^{r}(h)=\text{F}{\text{J}}_{q}^{r}(g)+\text{G}{\text{J}}_{q}^{r}(f),$$

where

3.9 $$\text{F}=\left(\begin{array}{cccc} f_{a} & -f_{b} & -f_{c} & -f_{d}\\ f_{b} & f_{a} & -f_{d} & f_{c}\\ f_{c} & f_{d} & f_{a} & -f_{b}\\ f_{d} & -f_{c} & f_{b} & f_{a} \end{array}\right)\;\mathrm{and}\;\text{G}=\left(\begin{array}{cccc} g_{a} & -g_{b} & -g_{c} & -g_{d}\\ g_{b} & g_{a} & g_{d} & -g_{c}\\ g_{c} & -g_{d} & g_{a} & g_{b}\\ g_{d} & g_{c} & -g_{b} & g_{a} \end{array}\right)$$

Upon multiplying both sides of (3.9) by **A** and (**A**^*μ*^)^−1^ and using lemma 2.6, we have

3.10 $$\begin{array}{rl} {\text{J}}_{q^{\mu }}^{a}(h)=\text{A}{\text{J}}_{q}^{r}(h){\left({\text{A}}^{\mu }\right)}^{-1} & =\text{A}\text{F}{\text{A}}^{-1}\text{A}{\text{J}}_{q}^{r}(g){\left({\text{A}}^{\mu }\right)}^{-1}+\text{A}\text{G}{\text{J}}_{q}^{r}(f){\left({\text{A}}^{\mu }\right)}^{-1}\\ & ={\text{F}}^{a}{\text{J}}_{q^{\mu }}^{a}{\left(g)+({\text{A}}^{T}{\text{G}}^{a}\right)}^{T}{\text{J}}_{q}^{r}(f){\left({\text{A}}^{\mu }\right)}^{-1}, \end{array}$$

where

3.11 $${\text{F}}^{a}≜{\text{AFA}}^{-1}=\left(\begin{array}{cccc} f & 0 & 0 & 0\\ 0 & f^{i} & 0 & 0\\ 0 & 0 & f^{j} & 0\\ 0 & 0 & 0 & f^{k} \end{array}\right)\;\mathrm{and}\;{\text{G}}^{a}≜\left(\begin{array}{cccc} g & 0 & 0 & 0\\ 0 & g^{i} & 0 & 0\\ 0 & 0 & g^{j} & 0\\ 0 & 0 & 0 & g^{k} \end{array}\right)$$

Upon extracting the first row of the matrix equation (3.10), we arrive at

3.12 $$\begin{array}{rl} {\left({\text{J}}_{q^{\mu }}^{a}(h)\right)}_{1} & =f{\left({\text{J}}_{q^{\mu }}^{a}(g)\right)}_{1}+{\left(\text{A}\right)}_{1}g{\text{J}}_{q}^{r}(f){\left({\text{A}}^{\mu }\right)}^{-1}=f{\left({\text{J}}_{q^{\mu }}^{a}(g)\right)}_{1}+{\left(\text{A}\right)}_{1}{\text{J}}_{q}^{r}(f)g{\left({\text{A}}^{\mu }\right)}^{-1}\\ & =f{\left({\text{J}}_{q^{\mu }}^{a}(g)\right)}_{1}+{\left(\text{A}\right)}_{1}{\text{J}}_{q}^{r}(f){\left({\text{A}}^{g\mu }\right)}^{-1}g=f{\left({\text{J}}_{q^{\mu }}^{a}(g)\right)}_{1}+{\left({\text{J}}_{q^{g\mu }}^{a}(f)\right)}_{1}g, \end{array}$$

where (**X**)_1_ denotes the first row of the matrix **X**. The first element of the above vector equation finally yields

3.13 $$\frac{\partial h}{\partial q^{\mu }}=f\frac{\partial g}{\partial q^{\mu }}+\frac{\partial f}{\partial q^{g\mu }}g.$$

This completes the proof of theorem 3.2. ▪

## 4. Application example

The WL-QLMS algorithm is based on the quaternion widely linear model *y*(*n*)=**w**^*T*^(*n*)**q**(*n*), where $\text{q}(n)={\left({\text{x}}^{T}(n),{\text{x}}^{iT}(n),{\text{x}}^{jT}(n),{\text{ x}}^{kT}(n)\right)}^{T}\in H^{4N}$ is the augmented input vector and $\text{w}(n)\in H^{4N}$ the associated weight (parameter) vector. The cost function to be minimized is a real-valued function of quaternion variables, given by

4.1 $$J(n)=|e(n){|}^{2}=|d(n)-y(n){|}^{2},$$

where *e*(*n*)=*d*(*n*)−*y*(*n*) is the error between the desired signal *d*(*n*) and the filter output *y*(*n*). The weight update of WL-QLMS is then given by [3]

4.2 $$\text{w}(n+1)=\text{w}(n)+\alpha e(n){\text{q}}^{*}(n),$$

where *α*>0 is the step size. Similar to the scalar duality in lemma 2.4, the duality between the augmented quaternion vector **q**(*n*) and its dual quadrivariate real vector $\text{r}(n)={\left({\text{x}}_{a}^{T}(n),{\text{x}}_{b}^{T}(n),{\text{x}}_{c}^{T}(n),{\text{x}}_{d}^{T}(n)\right)}^{T}\in R^{4N}$ is given by [7]

4.3 $$\text{q}(n)=\text{J}\text{r}(n),\;{\text{q}}^{H}(n)={\text{r}}^{H}(n){\text{J}}^{H}={\text{r}}^{T}(n){\text{J}}^{H},$$

where **J**=**A**⊗**I**_*N*_ denotes the Kronecker product of **A** (cf. (2.3)) and the *N*×*N* identity matrix **I**_*N*_. The filter output *y*(*n*) can now be rewritten as

4.4 $$y(n)={\text{w}}^{T}(n)\text{q}(n)={\text{w}}^{T}(n)\text{J}\text{r}(n)={\text{v}}^{T}(n)\text{r}(n),$$

where ${\text{v}}^{T}(n)={\text{w}}^{T}(n)\text{J}\in H^{4N}$ is an alternative augmented quaternion weight vector. Multiplying both sides of (4.2) by **J** and noting that **J**^*H*^**J**=4**I**_4*N*_, we have

4.5 $$\begin{array}{rl} {\text{w}}^{T}(n+1)\text{J} & ={\text{w}}^{T}(n)\text{J}+\alpha e(n){\text{q}}^{H}(n)\text{J}={\text{w}}^{T}(n)\text{J}+\alpha e(n){\text{r}}^{T}(n){\text{J}}^{H}\text{J}\\ & ={\text{w}}^{T}(n)\text{J}+4\alpha e(n){\text{r}}^{T}(n). \end{array}$$

Upon replacing **w**^*T*^(*n*)**J** with **v**^*T*^(*n*), the dual version of the WL-QLMS (D-WL-QLMS) algorithm becomes

4.6 v(n+1)=v(n)+αe(n)r(n),

where the constant 4 in (4.5) is absorbed into the step size *α*.

**Remark 4.1**

From (4.4), we can see that the D-WL-QLMS and WL-QLMS have the same filter output. Therefore, the D-WL-QLMS has the same performance as the WL-QLMS, which is better than the strictly linear QLMS and the real LMS (RLMS) [5,11], when dealing with non-circular inputs. Table 1 shows that D-WL-QLMS also has a lower computational complexity than the WL-QLMS, owing to the use of real-valued input vector **r**(*n*) in the calculations. This is similar to the operation of the RLMS; however, the weight vector **v**(*n*) of the D-WL-QLMS is quaternion-valued, which is different from the RLMS.

**Table 1.** Computational complexity in terms of real operations per iteration (*N* is the filter length).

algorithm	+	×
QLMS	32*N*	32*N*+4
WL-QLMS	128*N*	128*N*+4
D-WL-QLMS	32*N*	32*N*+4

**Remark 4.2**

Note that if we start from *y*(*n*)=**w**^*H*^(*n*)**q**(*n*), then an alternative form of the WL-QLMS is given by **w**(*n*+1)=**w**(*n*)+*α* **q**(*n*)e*(*n*) [3]. Denote **v**^*T*^(*n*)=**w**^*H*^(*n*)**J**, then the filter output becomes *y*(*n*)=**w**^*H*^(*n*)**Jr**(*n*)=**v**^*T*^(*n*)**r**(*n*), that is, the same as in (4.4). Finally, the proposed D-WL-QLMS keeps the same form of update rule in (4.6), and has the same computational complexity as that shown in table 1.

## 5. Conclusion

We have provided a new proof for the GHR calculus through the duality relationships between the GHR calculus and multivariate real calculus. These results complement the original proof given in [3], are easier to understand and are physically meaningful, and thus provide additional insights into the operation of the GHR calculus. An application example in adaptive learning theory demonstrates the advantages of the proposed approach.
